# Reproducibility of a 69-Item School Children’s Food Frequency Questionnaire (69-Item SCh-FFQ) and Diet Quality Scores Among Polish Children Aged 7–9 Years

**DOI:** 10.3390/nu18162712

**Published:** 2026-08-19

**Authors:** Kamila Sobaś, Joanna Kowalkowska, Edyta Suliga, Lidia Wadolowska

**Affiliations:** 1 Institute of Health Sciences, Medical College, Jan Kochanowski University, 5 Żeromskiego Street, 25-369 Kielce, Poland; kamila.sobas@ujk.edu.pl (K.S.); edyta.suliga@ujk.edu.pl (E.S.); 2 Department of Human Nutrition, Faculty of Food Science, University of Warmia and Mazury in Olsztyn, 45F Sloneczna Street, 10-718 Olsztyn, Poland; lidia.wadolowska@uwm.edu.pl; 3 Institute of Genetics and Animal Biotechnology, Polish Academy of Sciences, 36A Postepu Street, 05-552 Magdalenka, Poland

**Keywords:** dietary assessment, reliability, food consumption frequency, diet quality, school-aged children

## Abstract

**Background/Objectives**: Reliable dietary assessment tools are essential for evaluating dietary habits and diet quality in children. The aim of this study was to assess the reproducibility of a 69-item School Children’s Food Frequency Questionnaire (69-item SCh-FFQ) and the diet quality scores among Polish children aged 7–9 years. **Methods**: The reproducibility study was conducted among 77 children. The 69-item SCh-FFQ was administered twice (test and retest). Data on food consumption frequency were used to evaluate two diet quality scores: the pro-Healthy Diet Index (pHDI) and the non-Healthy Diet Index (nHDI). The reproducibility of the 69-item SCh-FFQ and the diet quality scores was evaluated using comparisons of mean values, cross-classification analysis, Cohen’s kappa statistic, Spearman’s rank correlation coefficient, intraclass correlation coefficient (ICC), and Bland–Altman analysis. **Results**: No significant differences were observed between test and retest of the FFQ for mean values of the pHDI (2.9 ± 1.8 vs. 2.8 ± 1.9 points; *p* = 0.124) or the nHDI (4.3 ± 1.5 vs. 4.2 ± 1.5 points; *p* = 0.611). Total agreement for the classification of children into categories of the diet quality scores reached 81.8% for pHDI and 75.3% for nHDI, with low gross misclassification rates (1.3% and 0.0%, respectively). Cohen’s kappa statistic indicated moderate to good agreement (κ = 0.68 for pHDI and κ = 0.51 for nHDI). For all food items, total agreement ranged from 66.2% to 94.8%, while the kappa statistic ranged from 0.56 to 0.81. Strong correlations and good agreement were observed between repeated measurements for both pHDI (r = 0.748, ICC = 0.778; *p* < 0.001) and nHDI (r = 0.783, ICC = 0.752; *p* < 0.001). Bland–Altman analysis demonstrated good agreement and no evidence of proportional bias. **Conclusions**: The 69-item SCh-FFQ demonstrated satisfactory reproducibility for assessing food consumption frequency and diet quality scores in children aged 7–9 years. Therefore, this questionnaire is a promising tool for future epidemiological studies among early school-aged children, although its utility should be confirmed in further validation studies using an appropriate reference method.

## 1. Introduction

An important part of an assessment of dietary habits is selecting the appropriate measurement methods, techniques, and tools. This choice depends on the aim of the study, as well as the required accuracy, availability of data sources, and associated costs. Food frequency questionnaires (FFQs) are considered the most feasible tools, particularly for epidemiological studies [1]. They are easy to apply, inexpensive, convenient for both researchers and respondents, and provide repeatable data within a relatively short time. The FFQ data can be used to monitor the dietary patterns of a population over time and to identify dietary habits that may require intervention. Due to cultural differences related to food availability and dietary preferences between populations, each newly developed FFQ should be validated for a target population. This is a necessary step before a given FFQ can be considered as a reliable research tool and applied for scientific and practical purposes. The first and fundamental stage in the validation of an FFQ is the assessment of its test–retest reproducibility [2,3,4,5].

An appropriate diet during childhood is crucial for the growth and development of children, allowing them to reach their full physical, cognitive, and emotional potential. It also significantly influences a population’s long-term health outcomes, including the risk of diet-related chronic diseases in adulthood [6]. However, there are few FFQs designed for younger school-age children, especially for those aged 7–9 years, as dietary assessment in this age group poses a challenge for researchers. The accuracy of self-reported food consumption by children may be limited due to their insufficient knowledge about food and difficulties in recalling the consumed foods. Consequently, studies involving children are usually conducted among their parents or guardians as proxy reporters, or at least with the participation of their parents or guardians [7,8]. Unfortunately, the parents or guardians of children who spend a part of the day at school may not have detailed knowledge about all of the foods consumed by their children. There is increasing evidence that children can provide reliable responses on issues relevant to them, as long as the questions are adapted to their cognitive abilities and developmental stage [9]. Therefore, research tools should be designed to match children’s needs and communication styles of children in order to facilitate their engagement in studies [10].

To date, relatively few questionnaires for early school-age children have been developed, especially in Central and Eastern European countries [7,11,12,13,14]. Political and economic transformations in these countries over the past several decades, along with ongoing computerisation and the increase in the number of cars in households (contributing to decreased physical activity), the expansion of supermarket chains and fast-food restaurants, and the widespread advertising of unhealthy foods in the mass media have contributed to rising rates of overweight and obesity among successive generations of children and adolescents [15,16]. Consequently, it is necessary to continuously monitor the diets of children in these countries using appropriate research tools. In Poland, comprehensive FFQs developed specifically for children remain limited. The SF-FFQ4PolishChildren^®^ is a short-form multicomponent dietary questionnaire, validated among Polish children and adolescents aged 6–15 years, that comprises only 9 key food groups and is therefore not intended to provide a comprehensive assessment of the overall habitual diet of school-aged children [7]. Furthermore, the reproducibility of an 85-item Child-FFQ, enabling a comprehensive assessment of diet quality, was evaluated in the PITNUTS 2024 study [17]; however, this tool was developed and validated for children aged 1–6 years. To date, no comprehensive FFQ enabling an assessment of the overall habitual diet of Polish children aged 7–9 years has been developed, despite this being a formative period for dietary habit development, with long-term implications for health. Therefore, the aim of this study was to assess the test–retest reproducibility of a 69-item School Children’s Food Frequency Questionnaire (69-item SCh-FFQ) and the diet quality scores among Polish children aged 7–9 years.

## 2. Materials and Methods

### 2.1. Study Design and Participants

The study was carried out with the consent of the Bioethics Committee at the Collegium Medicum of Jan Kochanowski University in Kielce, provided on 8 September 2023 (Approval Number: 48/2023). Informed consent was obtained from parents/legal guardians of children aged 7–9 years. During the study, all children were accompanied by their mothers. This study was conducted from March to September 2025. Potential participants were informed about the aims, scope, and organization of the study.

The study included two meetings with each respondent. At the first meeting, children were asked to complete the self-administered 69-item School Children’s Food Frequency Questionnaire (the 69-item SCh-FFQ) in paper form, and their mothers were asked a set of questions on family sociodemographics. At the second meeting (after two weeks), children completed the 69-item SCh-FFQ again (retest). Both administrations of the 69-item SCh-FFQ were completed by the children independently, under the supervision of a researcher, who was available to clarify general instructions or questions without influencing participants’ responses. Although the questionnaire instructions permitted children to ask a parent or another adult familiar with their eating habits for clarification if unsure how to report their consumption frequency for a specific food item, none of the participating children required such assistance. Furthermore, the two-week interval between the test and retest was selected based on previous methodological practice in studies assessing the reproducibility of food frequency questionnaires in children and adolescents [7,18]. This interval was considered sufficiently long to reduce the likelihood of recalling previous responses, while being short enough to minimize potential changes in habitual dietary intake.

**Figure 1:**
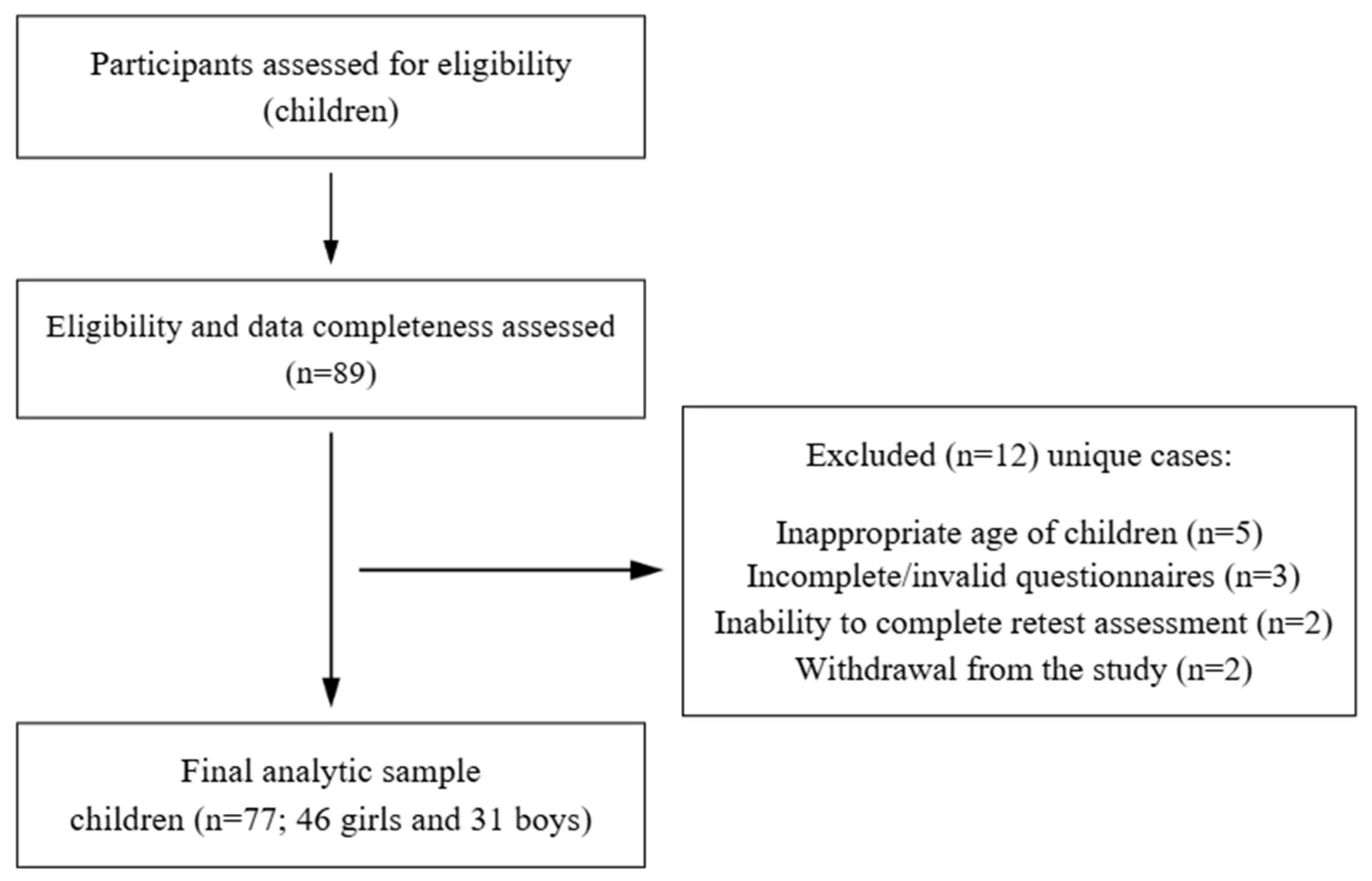
Flowchart of participant selection and inclusion in the final analytic sample. *n*—number of participants.

Children of the Świętokrzyskie Voivodeship were recruited through announcements distributed in schools. Schools and classes from both urban and rural areas were randomly selected. If approval from school authorities or parents/guardians was not obtained or if the response rate was low, additional schools were randomly selected. The final sample included 77 children who completed both administrations of the 69-item SCh-FFQ. Children who were outside the eligible age range or who did not complete the 69-item SCh-FFQ were excluded from the analysis. A flowchart illustrating participant selection and exclusions is presented in Figure 1.

### 2.2. The 69-Item School Children’s Food Frequency Questionnaire (69-Item SCh-FFQ)

**Figure 2:**
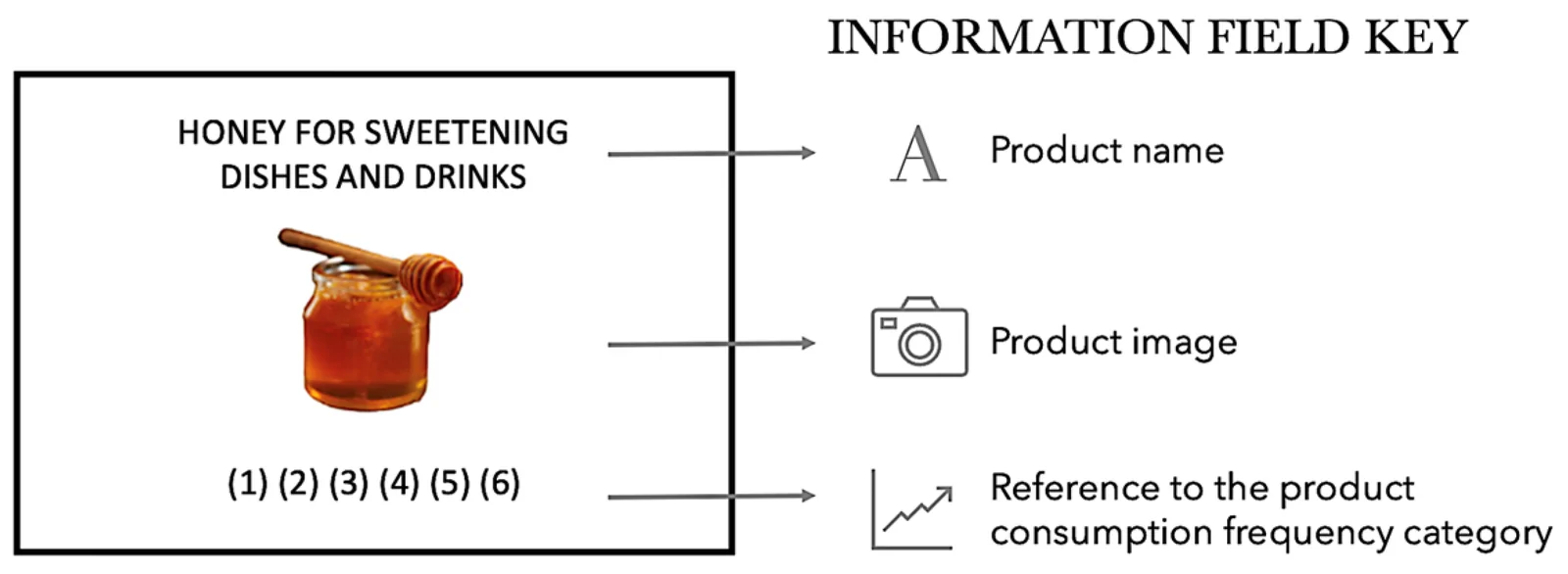
Questionnaire design. Numbers in parentheses indicate food consumption frequency categories: (1) never or almost never, (2) once a month or less frequently, (3) several times a month, (4) several times a week, (5) every day, (6) several times a day.

A pilot study involving 25 children aged 7–9 years was conducted to assess the comprehensibility, clarity, and suitability of the newly developed 69-item SCh-FFQ for children. During the pilot study, the researcher observed the children while they completed the questionnaire and assessed their understanding of the questions, food items, and graphical representations. The images in the questionnaire were used in accordance with the Freepik and Pixabay licences valid at the time of download or were created by the authors. This approach enabled children to easily recognize whether they consumed the given products (Figure 2). Cognitive interviewing was used to explore how children understood and interpreted the questions and response options. Most children were able to correctly recognize the food products presented in the illustrations, including products that they did not personally consume. This supported the suitability of the graphical representations for the target age group. The pilot testing did not result in major modifications to the content of the questionnaire. Minor improvements were introduced, mainly concerning the clarity of the graphical representations and selected language formulations.

The 69-item SCh-FFQ was developed by adapting the 72-item Semi-Quantitative Food Frequency Questionnaire (72-item SQ-FFQ), previously developed and validated among Polish young adults [19]. The 62-item non-quantitative Food Frequency Questionnaire (62-item FFQ-6^®^) [20] was additionally considered when defining and organising the food groups included in the questionnaire. Both previously developed questionnaires [19,20] were presented solely as text-based tables without any graphical support. In contrast, the final 69-item SCh-FFQ features both a modified food item list and a new graphical layout tailored to school-aged children. The final list of 69 items was obtained by adapting the 72-item SQ-FFQ to the dietary characteristics of school-aged children. Specifically, three items referring to alcoholic beverages were excluded, as they were not relevant to this population, and two items originally used to assess fruit and vegetable consumption in total were removed. Conversely, two items considered relevant for assessing beverage consumption among children were added: water, lemonade and so-called flavoured water.

### 2.3. Dietary Data Collection

The well-trained researchers instructed child-respondents in detail on how to complete the questionnaire. Each respondent reported habitual frequency of consumption of the food items by indicating one of the six categories, which were converted into daily frequency (times/day): never or almost never (0), once a month or less frequently (9/365 = 0.025), several times a month (3/30 = 0.1), several times a week (4/7 = 0.571), every day (1), several times a day (2). These conversion factors were selected a priori in accordance with the procedure established for the 62-item FFQ-6^®^ and the 72-item SQ-FFQ, which formed the basis for developing the 69-item SCh-FFQ and were developed and validated by Wądołowska’s research team [19,20]. This standardized approach was used to ensure consistency and comparability with these instruments and related studies.

Based on FFQ data, two diet quality scores were constructed: the pro-Healthy Diet Index (pHDI) and the non-Healthy Diet Index (nHDI). These indices were developed using predefined food groups and corresponding cut-off points for consumption frequency (i.e., one of the frequency of consumption categories from the FFQ). The pHDI included eight components: fruit, vegetables, pulses, nuts and seeds, wholegrain cereals, unsweetened dairy products, fish, and vegetable oils. The total pHDI score ranged from 0 to 8 points, with higher scores indicating greater adherence to a healthy dietary pattern. The nHDI consisted of nine components: sugar and sweets, salty snacks, sweetened dairy products, ready-to-eat breakfast cereals, sweet fruit preserves and candied fruits, fast food, processed meat, sweetened beverages, and energy drinks. The total nHDI score ranged from 0 to 9 points, with higher scores indicating greater adherence to an unhealthy dietary pattern. To calculate the total score for each diet quality index, first the daily frequencies of consumption of food items included in a given food group were summed (e.g., 8 food items constituted the “Fruit” component of the pHDI). If the sum of daily frequencies for a given food group met the criterion of consumption frequency specified for that group, 1 point was awarded. The consumption frequency criteria for each pHDI and nHDI component were derived from the six response options available in the 69-item SCh-FFQ, based on current dietary recommendations for children [21,22]. The components of both diet quality scores with consumption frequency criteria are presented in Table S2 (for pHDI) and Table S3 (for nHDI). Respondents were classified into one of three categories based on the total points for each diet quality index:

- The pHDI classification: 0–2 points—low, 3–5 points—moderate, 6–8 points—high level;
- The nHDI classification: 0–2 points—low, 3–5 points—moderate, 6–9 points—high level.

### 2.4. Other Variables

Anthropometric measurements (body weight and height) were taken during the first meeting by the researchers, according to the international guidelines [23]. The Body Mass Index-for-age (BMI-for-age) was calculated and the nutritional status of children was classified according to Polish growth references: underweight—below the 5th percentile; normal weight—from 5th percentile to less than the 85th percentile; overweight—from 85th percentile to less than the 95th percentile; and obesity—95th percentile or higher [24].

Sociodemographic data were collected through self-reported responses to closed-ended questions, except for age (calculated from the date of the research and the date of birth). Sociodemographic variables included sex, age (in years), place of residence (4 categories), and family economic situation (3 categories), and were based on standard questions from the KomPAN^®^ questionnaire validated among Polish adolescents and adults [25].

### 2.5. Statistical Analysis

Normality of continuous variables was tested using the Kolmogorov–Smirnov test. Mean values and standard deviations (SD) were calculated for continuous variables, and percentage distributions for categorical variables.

The reproducibility of the food consumption frequency and both diet quality scores (pHDI and nHDI) between the first and second administration of the 69-item SCh-FFQ was assessed using the Wilcoxon signed-rank test to compare mean values between test and retest. For food items showing statistically significant test–retest differences, effect sizes were additionally calculated as r = Z/√*N*, where Z is the test statistic from the Wilcoxon signed-rank test and *N* is the sample size, with r ≈ 0.10, 0.30 and 0.50 interpreted as small, medium and large effects, respectively [26,27].

Agreement between test and retest was further evaluated using cross-classification analysis and the unweighted Cohen’s kappa statistic, due to the non-linear distribution of the 6-point consumption frequency scale. In the cross-classification analysis, an acceptable level of total agreement was defined as more than 50% of children classified to the same category in test and retest [28]. Kappa values were interpreted as slight (0–0.20), fair (0.21–0.40), moderate (0.41–0.60), good (0.61–0.80) or excellent (0.81–1.00) [29]. Additionally, the kappa values greater than 0.40 were adopted as the threshold for acceptable agreement [28].

Test–retest reproducibility of the diet quality scores was also assessed using other statistics: Spearman’s rank correlation coefficient, the intraclass correlation coefficient (ICC) with 95% confidence interval (CI), and the Bland–Altman plots. The magnitude of Spearman’s correlation (r) was categorized as weak (0.00–0.29), moderate (0.30–0.49), good (0.50–0.69) or very good (0.70–1.00) [30]. The ICC was estimated using a two-way mixed-effects model with absolute agreement, based on single measurements, which is appropriate for evaluating test–retest reproducibility between two administrations of the FFQ, and was interpreted as poor (0–0.49), moderate (0.50–0.74), good (0.75–0.89) or excellent (0.90–1.00) [31]. Bland–Altman plots were constructed to graphically assess agreement for the pHDI and nHDI between test and retest of the 69-item SCh-FFQ. The *x*-axis represents the mean of the test and retest scores for each index, and the *y*-axis represents the difference between the two measurements [32]. The mean difference with 95% limits of agreement (LOA) was also presented on the plots. Internal reliability of the diet quality scores was assessed using Cronbach’s alpha [33].

A post hoc precision analysis showed that the final sample size (*n* = 77) was sufficient for a test–retest reproducibility study of the 69-item SCh-FFQ. Following Walter et al. [34], a sample of this size provides a 95% confidence interval (CI) width of ≤0.20 around an anticipated ICC of 0.75, which matches the high precision and narrow CIs observed for both diet quality scores (pHDI: ICC = 0.778, 95% CI: 0.671–0.853; nHDI: ICC = 0.752, 95% CI: 0.637–0.835).

Statistical analyses were performed using STATISTICA 13.3 (TIBCO Software Inc., Cracow, Poland) and PS IMAGO PRO 10.0, including IBM SPSS Statistics 29 (Predictive Solutions, Cracow, Poland).

## 3. Results

The study included 77 children aged 7–9 years. The mean age of the participants was 8.6 ± 0.7 years (Table 1). The study group consisted of 31 boys (40.3%) and 46 girls (59.7%). Most children lived in rural areas (54.5%). Most families reported an average economic status (85.7%). Considering the diet quality scores, 50.6% of children were classified as having a low level of pHDI, 40.3% a moderate level of pHDI, and 9.1% a high level of pHDI. For the nHDI, 11.7% of children had low level of this index, 66.2% of participants presented a moderate level, and 22.1% a high level of nHDI. Analysis of BMI-for-age categories showed that 45.4% of children had normal body weight, while 46.8% of children had excessive body weight—16.9% were classified as overweight and 29.9% were obese.

The reproducibility analysis of the 69-item SCh-FFQ demonstrated high stability of dietary quality indices and food consumption frequencies between the test and retest of the questionnaire (Table 2). Mean values of the dietary quality indices were very similar across both assessments. The mean pHDI score was 2.9 ± 1.8 points in the test and 2.8 ± 1.9 points in the retest (*p* = 0.124), whereas the nHDI score was 4.3 ± 1.5 and 4.2 ± 1.5 points, respectively (*p* = 0.611). These findings indicate good reproducibility of the questionnaire in assessing diet quality. For the majority of the 69 analyzed food items, no significant differences were observed between the test and retest of the 69-item SCh-FFQ, indicating reliable self-reported assessments of dietary intake over time. Statistically significant differences were identified for only five food items, as follows: wholemeal bread (0.30 ± 0.52 vs. 0.39 ± 0.56, *p* = 0.044), other animal fats (0.04 ± 0.13 vs. 0.08 ± 0.22, *p* = 0.015), processed meats such as wieners and hot dogs (0.43 ± 0.49 vs. 0.38 ± 0.47, *p* = 0.043), dried legumes (0.09 ± 0.29 vs. 0.09 ± 0.28, *p* = 0.047) and energy drinks (0.02 ± 0.09 vs. 0.01 ± 0.07, *p* = 0.039). All these items demonstrated small effect sizes, ranging from 0.22 to 0.27. Given their minor absolute magnitude, these discrepancies are likely of limited practical relevance in a dietary assessment context.

Total agreement for the classification of the children into categories of the dietary quality indices reached 81.8% for pHDI and 75.3% for nHDI (Table 3). At the same time, the percentage of gross misclassification (≥2 categories) was low, amounting to 1.3% and 0.0%, respectively. The kappa statistic indicated moderate to good agreement, with κ = 0.68 for pHDI and κ = 0.51 for nHDI.

For all 69 analyzed food items, the total agreement was acceptable (>50.0%), ranging from 66.2% to 94.8% (Table 3). The highest agreement was observed for food items consumed relatively rarely, i.e., game (94.8%), energy drinks (93.5%), water (88.3%), olives (88.3%), margarine (87.0%), and dried fruit (85.7%). The kappa statistic ranged from 0.56 to 0.81 for all food items, indicating acceptable agreement (>0.40). The highest kappa values were obtained for water (κ = 0.81), margarine (κ = 0.81), game (κ = 0.80), dried fruit (κ = 0.78), cured meats and offal products (κ = 0.78), and red meat (κ = 0.77). These findings indicate high classification consistency and good reproducibility of the reported intake frequency for these products.

Spearman’s correlations and ICC demonstrated high agreement between the first and second measurements for both the pro-Healthy Diet Index (pHDI) and the non-Healthy Diet Index (nHDI; Table 4). For the pHDI, a strong positive correlation was observed between the test and retest measurements (r = 0.748, *p* < 0.001) as well as good agreement (ICC = 0.778, *p* < 0.001), indicating high reproducibility of this diet quality index. For the nHDI, the Spearman correlation coefficient was slightly higher (r = 0.783, *p* < 0.001), while ICC was 0.752 (*p* < 0.001), confirming good agreement between repeated measurements. The internal reliability of the diet quality scores was assessed using Cronbach’s alpha coefficient (Tables S4 and S5). Cronbach’s alpha for the pHDI was 0.62, while for the nHDI it was 0.51.

**Figure 3:**
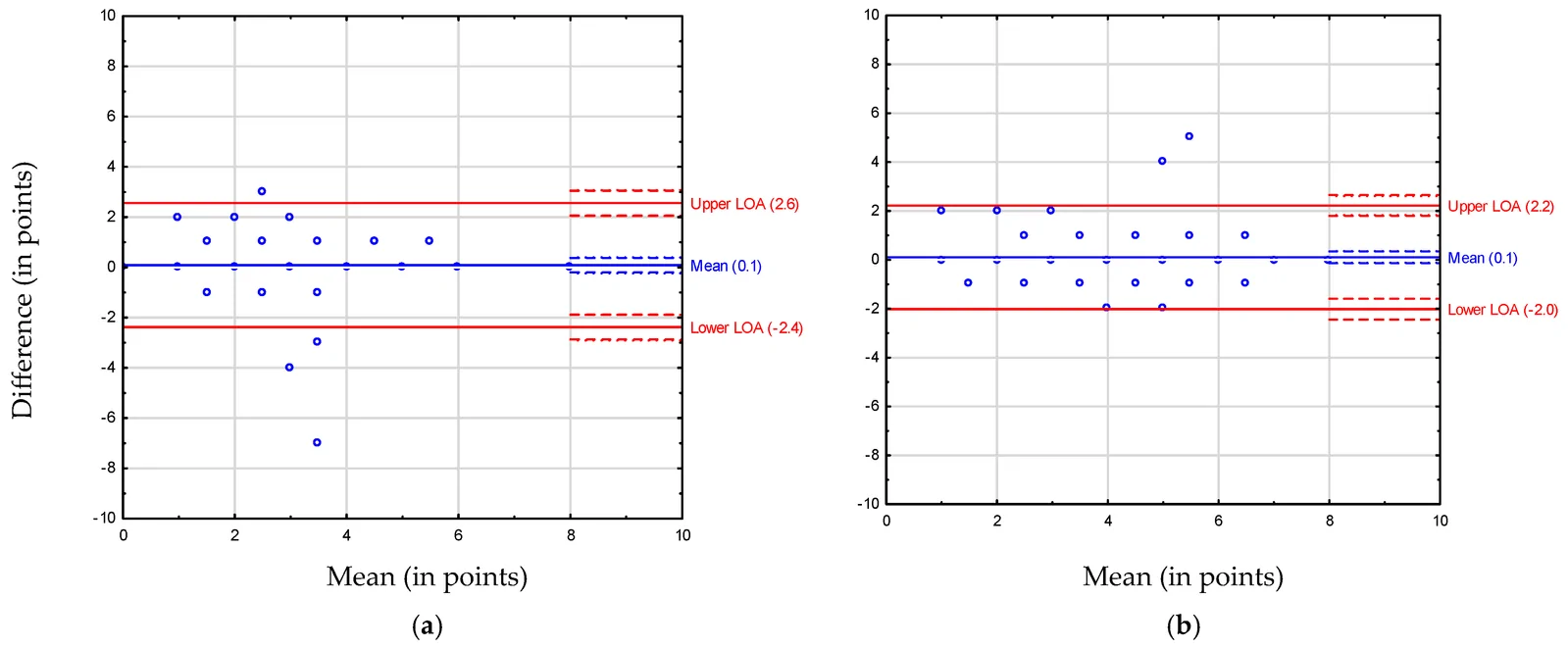
Bland–Altman plots for the diet quality scores for children aged 7–9 years old between the first and the second administration of the 69-item School Children’s Food Frequency Questionnaire (69-item SCh-FFQ): (**a**) the pro-Healthy Diet Index for children (pHDI), (**b**) the non-Healthy-Diet-Index for children (nHDI). Mean—mean difference between the first and the second administration of the 69-item SCh-FFQ (blue solid line) with 95% CI (dashed lines). LOA—95% limits of agreement between the first and the second administration of the 69-item SCh-FFQ (red solid lines) with 95% CI (dashed lines).

The Bland–Altman analysis demonstrated good agreement between the first and second administration of the 69-item SCh-FFQ for both diet quality scores (Figure 3). For the pHDI, the mean difference was 0.1 points and the 95% limits of agreement (LOA) ranged from −2.4 to 2.6 points. Similarly, for the nHDI, the mean difference was 0.1 points and the 95% LOA ranged from −2.0 to 2.2 points. Most observations were located within the 95% limits of agreement. No clear tendency toward increasing discrepancies with higher mean score of each diet quality index was observed, indicating the absence of proportional bias.

## 4. Discussion

The present study demonstrated satisfactory test–retest reproducibility of the 69-item SCh-FFQ among children aged 7–9 years, both in terms of consumption frequency of individual food items and the diet quality indices—the pro-healthy diet index (pHDI) and non-healthy diet index (nHDI)—which reflect a holistic approach to assessing children’s overall dietary patterns. These findings therefore support the usefulness of the 69-item SCh-FFQ for assessing diet quality and for monitoring dietary patterns among school-aged children.

The test–retest reproducibility findings observed in the present study are consistent with those reported for FFQs developed for school-aged children in other countries. For example, Qin et al. developed and evaluated a semi-quantitative FFQ among children aged 6–12 years in Western China [35]. The reproducibility analysis showed ICCs ranging from 0.20 to 0.85 for food items and from 0.37 to 0.75 for nutrients, indicating considerable variability across dietary components but generally acceptable reproducibility [35]. In a Croatian study among school-aged children aged 7–10 years, a semi-quantitative FFQ designed to assess fruit and vegetable consumption showed higher and more consistent reproducibility, with ICCs ranging from 0.724 to 0.826 [14]. Similarly, the recently developed Spanish COME-Kids F&B-FQ, evaluated among children aged 3–11 years, demonstrated substantial reproducibility, with mean ICCs of 0.64 for food groups and 0.62 for nutrients. The mean agreement between the two FFQ administrations was 86% for food groups and 85% for nutrients [36]. These findings are comparable to the high reproducibility observed for the 69-item SCh-FFQ and support the usefulness of FFQs for assessing habitual dietary patterns in children.

The agreement observed in the present study is also consistent with previous research showing that reproducibility may vary according to the type and frequency of food consumption. In the New Zealand study by Saeedi et al. among 9–10-year-old children, more than half of the analysed food items or groups had an ICC ≥ 0.50, while the median correlation between repeated FFQ administrations was 0.66 [18]. Similarly, in the present study, the highest consistency was generally observed for foods that are consumed routinely, whereas lower agreement was observed for some less frequently consumed products. This pattern may reflect the greater difficulty children have in recalling and estimating the usual frequency of consumption of foods that are consumed irregularly. Occasional consumption is more susceptible to random variation in dietary behaviour and recall error, particularly in younger children.

In the present study, statistically significant differences between the test and retest administrations were observed only for a few food items, including wholemeal bread, animal fats, processed meats, dried legumes, and energy drinks. However, these differences were small in absolute terms, suggesting limited practical relevance. Comparable variability across individual food groups has been reported in international reproducibility studies. The Chinese study, for example, demonstrated a wide range of ICC values across food items [35], while the Croatian and Spanish studies also showed differences in reproducibility depending on the food group assessed [14,36,37]. Such differences may reflect not only the properties of the questionnaire itself but also differences in habitual consumption patterns, cultural dietary practices, seasonality, and the frequency with which individual foods are consumed.

The results are also consistent with previous systematic evidence indicating that FFQs may provide more stable estimates for regularly consumed foods, whereas foods consumed sporadically tend to show lower agreement between repeated measurements [38]. The cognitive demands associated with estimating usual intake may be particularly relevant in children aged 7–9 years. In the present study, the questionnaire was specifically adapted to the cognitive abilities of this age group and included graphical representations of food products, which may have contributed to the high level of reproducibility observed.

Overall, the findings suggest that the 69-item SCh-FFQ provides stable measurements of food consumption frequency and diet quality indices in Polish children aged 7–9 years. The reproducibility estimates observed in the present study are within or above the range reported for several recently developed FFQs for children from other countries [35,36,37,39]. The good reproducibility of the 69-item SCh-FFQ has practical relevance because a stable dietary assessment tool is essential for epidemiological studies, dietary behaviour monitoring programmes, and assessments of diet quality in children [40,41]. However, reproducibility alone does not establish the validity of the questionnaire. Further studies comparing the 69-item SCh-FFQ with an independent dietary assessment method, such as repeated 24 h dietary recalls or dietary records, would be needed to determine its relative validity.

An analysis of the classification agreement demonstrated a high level of reliability of the 69-item SCh-FFQ for the assessment of diet quality scores and the frequency of consumption of specific food groups. The overall agreement for the diet quality scores amounted to 81.8% for the pHDI and 75.3% for the nHDI, with a very low percentage of gross misclassification. These results suggest that this questionnaire is a reliable tool for classifying children into diet quality categories, which is particularly relevant for epidemiological research and the monitoring of dietary behaviours. Similar levels of agreement were observed in the other FFQ validation study conducted among children and adolescents, in which the overall classification agreement mainly ranged between 70% and 85%, with a low percentage of incorrect classifications [38].

The overall agreement for all 69 food items analysed in this study was high, ranging from 66.2% to 94.8%, while the kappa statistic indicated moderate to very good agreement (κ = 0.56–0.81). These findings are consistent with the other study evaluating the reproducibility of FFQs in children, which also demonstrated a good stability of the responses and a good ability of the FFQ to classify respondents according to their food consumption frequency [35,36]. A particularly high agreement was noted for relatively rarely consumed foods, such as game meat, energy drinks and dried fruits. Similar results were reported in a study by Qin et al., who observed a higher repeatability of reporting consumption frequency for sporadically consumed food items than for regularly consumed ones, possibly because rare dietary behaviours are easier for the respondents to recall [35]. The high kappa coefficients obtained for water, margarine, dried fruit, red meat, cold cuts and offal products also suggest that products with clearly defined characteristics, or those consumed on specific occasions, are reported more consistently. These results match those observed in other FFQ validation study conducted among children and adolescents, which underlined that the repeatability of an assessment is usually higher for easily identifiable food groups than for products consumed every day in varying quantities [37].

The findings suggest that the 69-item SCh-FFQ has a good ability to classify respondents correctly into consumption categories and to minimise the risk of significant interpretative errors. This is particularly important in population studies, which apply FFQs primarily in order to rank the respondents according to their level of consumption or diet quality, rather than to quantify precisely the consumption of each product [38]. For both the pHDI and the nHDI, mean differences between the test and the retest were close to zero, indicating no proportional bias and good agreement between both administrations of this FFQ. The results obtained in this study are consistent with previous research on the reproducibility of FFQs among children, in which only minor differences were found between repeated measurements and a good agreement was observed between group-level assessments [39].

The results suggest that the 69-item SCh-FFQ may be a useful and reliable tool for assessing the children’s diet quality and classifying dietary patterns in epidemiological and intervention studies. The high classification agreement and the moderate to high kappa coefficients indicate good stability of the tool and its potential usefulness for monitoring dietary behaviours in the paediatric population [7,38].

### 4.1. Strengths and Limitations of the Study

The strengths of this study include the development of a 69-item SCh-FFQ adjusted to the cultural background of Polish children aged 7–9 years. The design of the questionnaire took into account the cognitive capabilities of early school-aged children. The tool includes colourful illustrations of the food items, which make it easier for the children to identify them and improve its attractiveness. During the study, it was observed that the graphical elements easily captured the children’s attention, sparked their interest, and promoted active participation in the assessment of their diet, despite the relatively long time needed to complete the questionnaire. A major advantage of this FFQ is its ability to assess food frequencies as well as determine diet quality scores, which increases the tool’s utility in epidemiological studies and monitoring programmes for children’s diets. It should be noted that the time interval between the first and second administrations of the FFQ was chosen in order to minimise the effect of participants remembering their previous responses and, at the same time, to reduce the likelihood of actual changes occurring in the children’s dietary habits. The reliability of the results may also have been improved by the fact that the study was conducted with the assistance of trained dietitians, who ensured that the procedure was carried out correctly and helped the participants to understand the questions in the FFQ.

On the other hand, the study has some limitations that should be highlighted. First, the analyses were conducted among a relatively small group of 77 children aged 7–9 years recruited from a single geographical region, which may limit the generalisability of the findings to the broader population of Polish children, as well as to those in other age groups or living in regions with different dietary patterns. However, the literature suggests a minimal sample size for an agreement analysis of about 50–100 persons [42], and a post hoc precision analysis confirmed that our final sample provided adequate statistical precision for the reliability estimates [34]. Secondly, consideration must be given to the potential influence of seasonality on dietary intake. The study was conducted between March and September, a period during which the availability and consumption of certain items, particularly fresh fruits and vegetables, fluctuate. Consequently, these shifts in dietary patterns may have influenced the reported food intake and the resulting diet quality scores. However, because all participants were assessed within the same timeframe, the impact of seasonal variation on the reproducibility of the SCh-FFQ and the indices was likely minimized. Another inherent constraint arises from the fact that the dietary data in the FFQ are self-reported, which involved a risk of memory errors and the underestimation or overestimation of consumption frequency of each food item. Furthermore, the present study assessed the reproducibility of the 69-item SCh-FFQ and the diet quality scores, but did not evaluate their criterion validity against a reference dietary assessment method to confirm that the tool accurately captures true dietary intake. Regarding the statistical properties of the indices, Cronbach’s alpha was 0.62 for the pHDI and 0.51 for the nHDI. Although these values are below the commonly recommended threshold of 0.70, they are comparable to those reported for other diet quality scores derived from FFQ data, where a lower internal consistency is often observed due to the heterogeneous nature of the dietary components and the limited number of items in each index [33]. Cronbach’s alpha was reported mainly for comparability with previously published diet quality indices, although its relevance for formative dietary indices is limited. Diet quality indices are better understood as formative constructs, in which components jointly define the score without being expected to correlate strongly [43]. Despite this modest internal consistency, both diet quality scores showed good test–retest reproducibility (ICC > 0.75). Additionally, confidence intervals for Cohen’s kappa were not reported, which limits the assessment of the precision of the agreement estimates obtained.

### 4.2. Implications for Future Research

Due to the dynamic changes in children’s dietary patterns and the growing significance of monitoring diet quality in Central and Eastern European countries. It is also necessary to further develop and apply appropriate research tools that allow regular assessment of children’s diets. Future studies should include larger and more diverse samples, recruited from multiple Polish regions, to confirm the results across different populations. It is important to conduct validation studies of the 69-item SCh-FFQ using reference methods such as multiple 24 h dietary recalls or food records. It is also advisable to evaluate the ability of the questionnaire to identify the relationships between diet quality and biomarkers of children’s health. Future studies should verify the usefulness of the tool for the assessment of associations between the results obtained with this FFQ and the nutritional status of children. This would allow for a comprehensive confirmation of the FFQ’s applicability in both scientific research and public health practice.

## 5. Conclusions

The 69-item SCh-FFQ demonstrated satisfactory test–retest reproducibility for assessing habitual food frequency and diet quality among children aged 7–9 years. These findings suggest that it may be a useful tool for classifying children according to their diet quality and for monitoring dietary patterns in this age group. However, further validation of the 69-item SCh-FFQ against an appropriate dietary reference method and relevant biomarkers is needed to confirm its suitability for use in epidemiological research in paediatric populations.

## Figures and Tables

**Table 1:** Participant characteristics (*n* = 77).

Variables	Children Aged 7–9 Years Old	
	*n*	%
Sample size	77	100
Sex		
boys	31	40.3
girls	46	59.7
Age (years) ^1^	8.6 ± 0.7 (7.1–9.9)	
Place of residence		
village	42	54.5
town < 20,000 inhabitants	8	10.4
town 20,000–100,000 inhabitants	6	7.8
city > 100,000 inhabitants	21	27.3
Economic situation of family		
below average	1	1.3
average	66	85.7
above average	10	13.0
pHDI category ^2^		
low	39	50.6
moderate	31	40.3
high	7	9.1
nHDI category ^3^		
low	9	11.7
moderate	51	66.2
high	17	22.1
BMI category ^4^		
underweight	6	7.8
normal weight	35	45.4
overweight	13	16.9
obesity	23	29.9

^1^ mean value ± standard deviation (min-max); ^2^ pHDI—pro-Healthy Diet Index for children aged 7–9 years old (low: 0–2, moderate: 3–5, high: 6–8 points); ^3^ nHDI—non-Healthy Diet Index for children aged 7–9 years old (low: 0–2, moderate: 3–5, high: 6–9 points); ^4^ BMI-for category—the age-specific Body Mass Index was calculated using measured height and weight and classified according to the Polish growth references: underweight—less than the 5th percentile; normal weight—5th percentile to less than the 85th percentile; overweight—85th percentile to less than the 95th percentile; obesity—95th percentile or higher [24].

**Table 2:** Mean values and standard deviations (SD) of the diet quality scores and food consumption frequency in test and retest of the 69-item SCh-FFQ (*n* = 77).

FFQ Item No.	Food Items	Test	Retest	*p*
		Mean (SD)	Mean (SD)	
	Diet quality scores (in points)			
	pHDI ^1^	2.9 (1.8)	2.8 (1.9)	0.124
	nHDI ^2^	4.3 (1.5)	4.2 (1.5)	0.611
	Food consumption frequency (times/day) ^3^			
1	Sugar for sweetening drinks	0.43 (0.52)	0.41 (0.51)	0.553
2	Honey for sweetening dishes and drinks	0.17 (0.37)	0.16 (0.37)	0.899
3	Chocolate, chocolate candies and bars	0.46 (0.42)	0.41 (0.41)	0.062
4	Non-chocolate candies	0.40 (0.45)	0.37 (0.41)	0.329
5	Biscuits and cakes	0.32 (0.41)	0.33 (0.42)	0.547
6	Ice creams and custard	0.49 (0.55)	0.47 (0.53)	0.850
7	Salty snacks	0.29 (0.33)	0.30 (0.33)	0.749
8	Milk and natural milk drinks	0.49 (0.51)	0.51 (0.57)	0.750
9	Sweetened milk and milk drinks	0.28 (0.46)	0.29 (0.46)	0.787
10	Natural curd cheeses	0.23 (0.44)	0.24 (0.44)	0.457
11	Flavoured smooth cheese	0.24 (0.49)	0.23 (0.49)	0.852
12	Cheeses	0.50 (0.54)	0.48 (0.49)	0.716
13	Eggs and egg dishes	0.58 (0.47)	0.61 (0.49)	0.529
14	Wholemeal breads	0.30 (0.52)	0.39 (0.56)	0.044
15	White breads	0.87 (0.49)	0.83 (0.44)	0.627
16	Coarse grain groats, unrefined	0.22 (0.40)	0.26 (0.49)	0.243
17	Fine grain groats, refined	0.20 (0.36)	0.18 (0.33)	0.645
18	Breakfast cereals	0.20 (0.44)	0.21 (0.40)	0.672
19	Ready-to-eat breakfast cereal products	0.54 (0.52)	0.55 (0.54)	0.969
20	Regular pasta (dishes)	0.56 (0.53)	0.52 (0.51)	0.271
21	Wholegrain pasta (dishes)	0.30 (0.40)	0.33 (0.39)	0.210
22	Sweet dumplings, pierogis and pancakes	0.33 (0.38)	0.34 (0.39)	0.972
23	Salty dumplings, pierogis and pancakes	0.15 (0.36)	0.13 (0.22)	0.474
24	Fast-food dishes	0.17 (0.24)	0.19 (0.27)	0.716
25	Oil	0.41 (0.39)	0.37 (0.39)	0.179
26	Butter	0.75 (0.59)	0.67 (0.57)	0.124
27	Margarine	0.20 (0.44)	0.21 (0.44)	0.442
28	Cream	0.29 (0.51)	0.27 (0.51)	0.436
29	Other animal fats	0.04 (0.13)	0.08 (0.22)	0.015
30	Mayonnaise and salad dressings	0.33 (0.53)	0.30 (0.49)	0.550
31	Stone fruit	0.59 (0.62)	0.51 (0.49)	0.168
32	Kiwi and citrus fruit	0.41 (0.44)	0.42 (0.39)	0.662
33	Tropical fruits	0.35 (0.49)	0.43 (0.54)	0.074
34	Berries	0.65 (0.55)	0.66 (0.53)	0.500
35	Bananas	0.63 (0.51)	0.62 (0.47)	0.958
36	Apples and pears	0.67 (0.48)	0.63 (0.43)	0.406
37	Avocados	0.17 (0.43)	0.13 (0.37)	0.213
38	Olives	0.07 (0.33)	0.01 (0.07)	0.065
39	Dried fruit	0.18 (0.44)	0.19 (0.41)	0.928
40	Sweet fruit preserves and candied fruit	0.33 (0.53)	0.26 (0.46)	0.110
41	Wieners, luncheon, meat, hot dogs	0.43 (0.49)	0.38 (0.47)	0.043
42	Sausages (various types)	0.28 (0.44)	0.28 (0.40)	0.587
43	High-quality cooked meats	0.52 (0.47)	0.53 (0.42)	0.439
44	Cured meats and offal products	0.22 (0.41)	0.27 (0.43)	0.050
45	Red meats	0.38 (0.53)	0.37 (0.54)	0.728
46	Poultry and rabbit meat	0.44 (0.40)	0.41 (0.41)	0.161
47	Game	0.01 (0.07)	0.04 (0.17)	0.066
48	Lean fish	0.20 (0.41)	0.18 (0.35)	0.944
49	Oily fish	0.14 (0.35)	0.18 (0.41)	0.068
50	Cruciferous vegetables	0.24 (0.46)	0.28 (0.41)	0.050
51	Yellow and orange-coloured vegetables	0.37 (0.47)	0.40 (0.43)	0.333
52	Green leafy vegetables	0.36 (0.48)	0.35 (0.41)	0.667
53	Tomatoes	0.56 (0.64)	0.51 (0.59)	0.704
54	Vegetables such as, e.g., cucumber, pumpkin	0.35 (0.40)	0.34 (0.36)	0.848
55	Root and other vegetables	0.36 (0.54)	0.36 (0.45)	0.835
56	Fresh legume seeds	0.23 (0.43)	0.22 (0.42)	0.899
57	Dried legume seeds	0.09 (0.29)	0.09 (0.28)	0.047
58	Potatoes	0.59 (0.42)	0.60 (0.43)	0.569
59	French fries, potato pancakes	0.38 (0.46)	0.35 (0.44)	0.522
60	Potato dishes	0.28 (0.48)	0.28 (0.41)	0.370
61	Nuts	0.27 (0.46)	0.18 (0.32)	0.097
62	Nut spreads	0.20 (0.38)	0.18 (0.34)	0.285
63	Seeds	0.14 (0.31)	0.13 (0.30)	0.526
64	Fruit juices and nectars	0.52 (0.52)	0.48 (0.48)	0.324
65	Vegetable and vegetable-fruit juices	0.24 (0.46)	0.25 (0.42)	0.091
66	Energy drinks	0.02 (0.09)	0.01 (0.07)	0.039
67	Sweetened drinks (sparkling and still)	0.34 (0.53)	0.33 (0.53)	0.710
68	Water	1.34 (0.66)	1.42 (0.64)	0.082
69	Lemonade and so-called flavoured water	0.46 (0.56)	0.44 (0.51)	0.775

SD—standard deviation; *p*—significance level of Wilcoxon signed-rank test; ^1^ pHDI—pro-Healthy Diet Index for children aged 7–9 years old (low: 0–2, moderate: 3–5, high: 6–8 points); ^2^ nHDI—non-Healthy Diet Index for children aged 7–9 years old (low: 0–2, moderate: 3–5, high: 6–9 points); ^3^ daily consumption frequency (range: 0–2 times/day).

**Table 3:** Classification agreement (%) and kappa statistic for the diet quality scores and food consumption frequency in test and retest of the 69-item SCh-FFQ (*n* = 77).

FFQ Item No.	Food Items	Total Agreement	Misclassification		Kappa
			±1 Category	±2 or More Categories	
	Diet quality scores				
	pHDI ^1^	81.8	16.9	1.3	0.68
	nHDI ^2^	75.3	24.7	0.0	0.51
	Food consumption frequency ^3^				
1	Sugar for sweetening drinks	77.9	10.4	11.7	0.73
2	Honey for sweetening dishes and drinks	81.8	9.1	9.1	0.75
3	Chocolate, chocolate candies and bars	75.3	20.8	3.9	0.66
4	Non-chocolate candies	81.8	11.7	6.5	0.75
5	Biscuits and cakes	77.9	15.6	6.5	0.70
6	Ice creams and custard	68.8	27.3	3.9	0.59
7	Salty snacks	74.0	19.5	6.5	0.66
8	Milk and natural milk drinks	66.2	23.4	10.4	0.58
9	Sweetened milk and milk drinks	67.5	22.1	10.4	0.59
10	Natural curd cheeses	80.5	6.5	13.0	0.74
11	Flavoured smooth cheese	74.0	13.0	13.0	0.62
12	Cheeses	79.2	13.0	7.8	0.73
13	Eggs and egg dishes	81.8	10.4	7.8	0.76
14	Wholemeal breads	68.8	11.7	19.5	0.60
15	White breads	74.0	11.7	14.3	0.59
16	Coarse grain groats, unrefined	79.2	11.7	9.1	0.73
17	Fine grain groats, refined	76.6	15.6	7.8	0.68
18	Breakfast cereals	71.4	11.7	16.9	0.61
19	Ready-to-eat breakfast cereal products	66.2	22.1	11.7	0.56
20	Regular pasta (dishes)	77.9	19.5	2.6	0.69
21	Wholegrain pasta (dishes)	75.3	11.7	13.0	0.68
22	Sweet dumplings, pierogis and pancakes	72.7	20.8	6.5	0.62
23	Salty dumplings, pierogis and pancakes	72.7	13.0	14.3	0.62
24	Fast-food dishes	75.3	13.0	11.7	0.64
25	Oil	72.7	19.5	7.8	0.65
26	Butter	70.1	19.5	10.4	0.61
27	Margarine	87.0	7.8	5.2	0.81
28	Cream	79.2	15.6	5.2	0.73
29	Other animal fats	83.1	9.1	7.8	0.62
30	Mayonnaise and salad dressings	79.2	15.6	5.2	0.74
31	Stone fruit	75.3	15.6	9.1	0.69
32	Kiwi and citrus fruit	76.6	11.7	11.7	0.70
33	Tropical fruits	76.6	13.0	10.4	0.71
34	Berries	79.2	11.7	9.1	0.73
35	Bananas	72.7	14.3	13.0	0.64
36	Apples and pears	79.2	13.0	7.8	0.71
37	Avocados	77.9	16.9	5.2	0.65
38	Olives	88.3	3.9	7.8	0.68
39	Dried fruit	85.7	7.8	6.5	0.78
40	Sweet fruit preserves and candied fruit	71.4	16.9	11.7	0.64
41	Wieners, luncheon, meat, hot dogs	79.2	11.7	9.1	0.73
42	Sausages (various types)	80.5	13.0	6.5	0.75
43	High-quality cooked meats	68.8	18.2	13.0	0.59
44	Cured meats and offal products	83.1	9.1	7.8	0.78
45	Red meats	81.8	14.3	3.9	0.77
46	Poultry and rabbit meat	79.2	13.0	7.8	0.71
47	Game	94.8	1.3	3.9	0.80
48	Lean fish	83.1	9.1	7.8	0.76
49	Oily fish	71.4	16.9	11.7	0.60
50	Cruciferous vegetables	75.3	10.4	14.3	0.69
51	Yellow and orange-coloured vegetables	80.5	10.4	9.1	0.75
52	Green leafy vegetables	68.8	19.5	11.7	0.61
53	Tomatoes	77.9	11.7	10.4	0.73
54	Vegetables such as, e.g., cucumber, pumpkin	72.7	9.1	18.2	0.65
55	Root and other vegetables	79.2	11.7	9.1	0.74
56	Fresh legume seeds	81.8	13.0	5.2	0.76
57	Dried legume seeds	84.4	7.8	7.8	0.74
58	Potatoes	83.1	11.7	5.2	0.76
59	French fries, potato pancakes	80.5	16.9	2.6	0.72
60	Potato dishes	76.6	15.6	7.8	0.70
61	Nuts	72.7	16.9	10.4	0.65
62	Nut spreads	75.3	14.3	10.4	0.67
63	Seeds	81.8	10.4	7.8	0.74
64	Fruit juices and nectars	79.2	10.4	10.4	0.73
65	Vegetable and vegetable-fruit juices	77.9	10.4	11.7	0.72
66	Energy drinks	93.5	1.3	5.2	0.70
67	Sweetened drinks (sparkling and still)	80.5	16.9	2.6	0.76
68	Water	88.3	9.1	2.6	0.81
69	Lemonade and so-called flavoured water	80.5	13.0	6.5	0.75

^1^ pHDI—pro-Healthy Diet Index for children aged 7–9 years old (low: 0–2, moderate: 3–5, high: 6–8 points); ^2^ nHDI—non-Healthy Diet Index for children aged 7–9 years old (low: 0–2, moderate: 3–5, high: 6–9 points); ^3^ daily consumption frequency (range: 0–2 times/day).

**Table 4:** Spearman’s correlation coefficient (r) and intraclass correlation coefficient (ICC) with 95% confidence interval (CI) for the diet quality scores in test and retest of the 69-item SCh-FFQ (*n* = 77).

Diet Quality Scores	r	*p*	ICC (95% CI)	*p*
pHDI ^1^	0.748	<0.001	0.778 (0.671–0.853)	<0.001
nHDI ^2^	0.783	<0.001	0.752 (0.637–0.835)	<0.001

^1^ pHDI—pro-Healthy Diet Index for children aged 7–9 years old (low: 0–2, moderate: 3–5, high: 6–8 points); ^2^ nHDI—non-Healthy Diet Index for children aged 7–9 years old (low: 0–2, moderate: 3–5, high: 6–9 points).

## Data Availability

Data presented in this study are available upon request from the first author.

## Supplementary Materials

The following supporting information can be downloaded at https://www.mdpi.com/article/10.3390/nu18162712/s1. Table S1. The 69-item School Children’s Food Frequency Questionnaire (69-item SCh-FFQ); Table S2. Components of the pro-Healthy Diet Index for children aged 7–9 years old (pHDI); Table S3. Components of the non-Healthy Diet Index for children aged 7–9 years old (nHDI); Table S4. Internal reliability of the pro-Healthy Diet Index for children aged 7–9 years old (pHDI) (*n* = 77); Table S5. Internal reliability of the non-Healthy Diet Index for children aged 7–9 years old (nHDI) (*n* = 77).

## Abbreviations

The following abbreviations are used in this manuscript:

BMI	Body Mass Index
FFQ	Food Frequency Questionnaire
ICC	Intraclass Correlation Coefficient
LOA	Limits of Agreement
nHDI	non-Healthy Diet Index
pHDI	pro-Healthy Diet Index
SD	Standard Deviation
62-item FFQ-6^®^	Reproducibility of a non-quantitative Food Frequency Questionnaire
69-item SCh-FFQ	69-item School Children’s Food Frequency Questionnaire
72-Item SQ-FFQ	72-Item Semi-Quantitative Food Frequency Questionnaire
