# Supplementary material for: Reproducibility of a 69-Item School Children’s Food Frequency Questionnaire (69-Item SCh-FFQ) and Diet Quality Scores Among Polish Children Aged 7–9 Years
Source: Nutrients. 2026 Aug 19;18(16):2712. doi: 10.3390/nu18162712 (PMC13516586; doi:10.3390/nu18162712)
Supplement: Supplementary file 1 [file nutrients-18-02712-s001.zip › nutrients-4444504-supplementary.pdf]

Supplementary materials

Table S1. The 69-item School Children's Food Frequency Questionnaire (69-item SCh-FFQ)

Food and drink consumption frequency questionnaire for school-age children

*Answer all the questions as best as you can. If you are unsure how to respond, provide an approximate answer or ask your parent or an adult who knows your eating habits. Choose **one** answer that best describes your eating pattern over the past 12 months. Include all meals and snacks eaten both at home and outside the home.*

Over the past 12 months, how often have you eaten:	Never or almost never	Once a month or less frequently	Several times a month	Several times a week	Every day	Several times a day
Consumption frequency codes	(1)	(2)	(3)	(4)	(5)	(6)
**Sweets and snacks**						

SUGAR FOR SWEETENING DRINKS<br>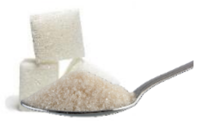<br>(1) (2) (3) (4) (5) (6)	HONEY FOR SWEETENING DISHES AND DRINKS<br>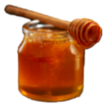<br>(1) (2) (3) (4) (5) (6)	CHOCOLATE, CHOCOLATE CANDIES AND BARS<br>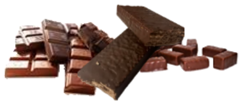<br>(1) (2) (3) (4) (5) (6)	NON-CHOCOLATE CANDIES<br>e.g. fruit candies, lollipops, jelly sweets, toffee<br>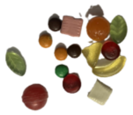<br>(1) (2) (3) (4) (5) (6)
BISCUITS AND CAKES<br>e.g. muffins, croissants, cream cakes, doughnuts<br>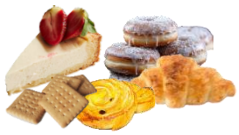<br>(1) (2) (3) (4) (5) (6)	ICE CREAM AND CUSTARD<br>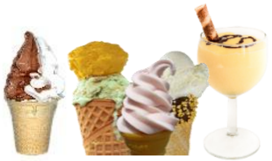<br>(1) (2) (3) (4) (5) (6)	SALTY SNACKS<br>e.g. crackers, crisps, pretzel sticks<br>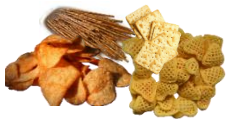<br>(1) (2) (3) (4) (5) (6)	

Over the past 12 months, how often have you drunk or eaten:	Never or almost never	Once a month or less frequently	Several times a month	Several times a week	Every day	Several times a day
Consumption frequency codes	(1)	(2)	(3)	(4)	(5)	(6)
**Dairy products and eggs**						

MILK AND NATURAL MILK DRINKS<br>e.g. yoghurt, kefir, buttermilk<br>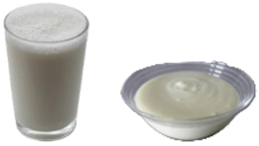<br>(1) (2) (3) (4) (5) (6)	SWEETENED MILK AND MILK DRINKS<br>e.g. fruit yoghurt, flavoured buttermilk<br>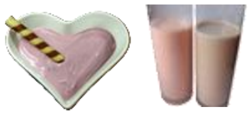<br>(1) (2) (3) (4) (5) (6)	NATURAL CURD CHEESES<br>e.g. curd cheeses, mozzarella<br>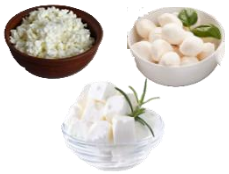<br>(1) (2) (3) (4) (5) (6)	FLAVOURED SMOOTH CHEESES<br>e.g. fruit-, vanilla-, chocolate-flavoured<br>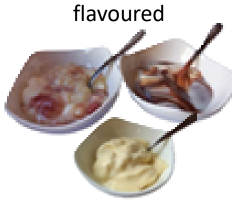<br>(1) (2) (3) (4) (5) (6)
CHEESES<br>e.g. hard cheeses, blue cheeses, processed cheeses, cheese spreads<br>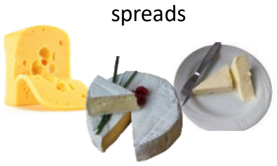<br>(1) (2) (3) (4) (5) (6)	EGGS AND EGG DISHES<br>e.g. omelette, scrambled eggs, egg paste<br>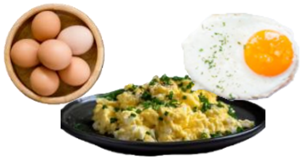<br>(1) (2) (3) (4) (5) (6)		

Over the past 12 months, how often have you eaten:	Never or almost never	Once a month or less frequently	Several times a month	Several times a week	Every day	Several times a day
Consumption frequency codes	(1)	(2)	(3)	(4)	(5)	(6)
**Cereal products**						

WHOLEMEAL BREADS<br>e.g. graham bread, wholemeal rye bread, seeded rolls<br>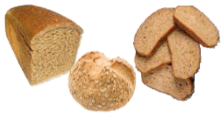<br>(1) (2) (3) (4) (5) (6)	WHITE BREADS<br>Kaiser rolls, toast bread, white wheat bread<br>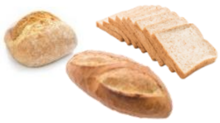<br>(1) (2) (3) (4) (5) (6)	COARSE GRAIN GROATS, UNREFINED<br>e.g. buckwheat groats, pearl barley, brown rice<br>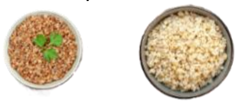<br>(1) (2) (3) (4) (5) (6)	FINE GRAIN GROATS, REFINED<br>e.g. white rice, semolina<br>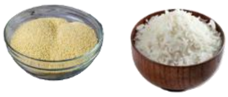<br>(1) (2) (3) (4) (5) (6)
BREAKFAST CEREALS<br>e.g. barley flakes, oatmeal, natural muesli<br>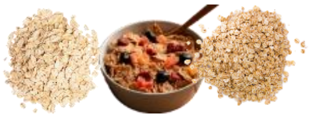<br>(1) (2) (3) (4) (5) (6)	READY-TO-EAT BREAKFAST CEREAL PRODUCTS<br>e.g. crunchy muesli, cornflakes, sweetened cereals<br>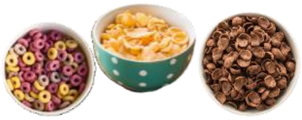<br>(1) (2) (3) (4) (5) (6)	REGULAR PASTA (DISHES)<br>e.g. spaghetti with sauce<br>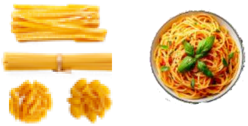<br>(1) (2) (3) (4) (5) (6)	WHOLEGRAIN PASTA (DISHES)<br>e.g. with tomato sauce<br>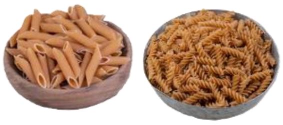<br>(1) (2) (3) (4) (5) (6)
SWEET DUMPLINGS, PIEROGIS AND PANCAKES<br>e.g. with fruit, cottage cheese<br>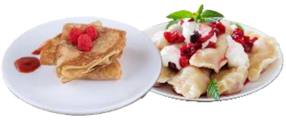<br>(1) (2) (3) (4) (5) (6)	SALTY DUMPLINGS, PIEROGIS AND PANCAKES<br>eg. with sauerkraut, spinach<br>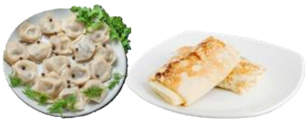<br>(1) (2) (3) (4) (5) (6)	FAST-FOOD DISHES<br>e.g. pizza, baked sandwiches, burgers, doner kebab<br>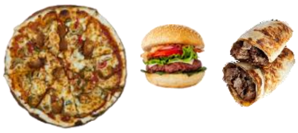<br>(1) (2) (3) (4) (5) (6)	

Over the past 12 months, how often have you drunk or eaten:	Never or almost never	Once a month or less frequently	Several times a month	Several times a week	Every day	Several times a day
Consumption frequency codes	(1)	(2)	(3)	(4)	(5)	(6)
**Fats**						

OIL<br>e.g. rapeseed oil, sunflower oil, olive oil<br>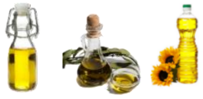<br>(1) (2) (3) (4) (5) (6)	BUTTER<br>e.g. in a block or tub<br>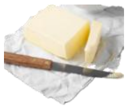<br>(1) (2) (3) (4) (5) (6)	MARGARINE<br>e.g. in a block or tub<br>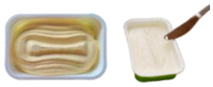<br>(1) (2) (3) (4) (5) (6)	CREAM<br>e.g. sour cream or whipping cream<br>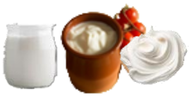<br>(1) (2) (3) (4) (5) (6)
OTHER ANIMAL FATS<br>e.g. bacon, lard<br>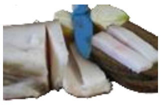<br>(1) (2) (3) (4) (5) (6)	MAYONNAISE AND SALAD DRESSINGS<br>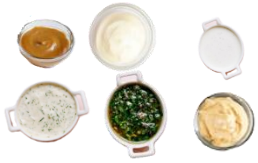<br>(1) (2) (3) (4) (5) (6)		

Over the past 12 months, how often have you eaten:	Never or almost never	Once a month or less frequently	Several times a month	Several times a week	Every day	Several times a day
Consumption frequency codes	(1)	(2)	(3)	(4)	(5)	(6)
**Fruit**						

STONE FRUITS
e.g. plums, apricots, sour or sweet cherries, grapes

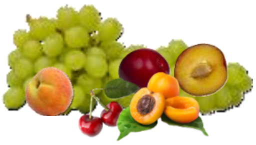

(1) (2) (3) (4) (5) (6)

KIWIS AND CITRUS FRUITS
e.g. grapefruits, pomelos, oranges, tangerines

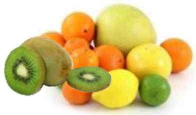

(1) (2) (3) (4) (5) (6)

TROPICAL FRUIT
e.g. mangoes, pineapples, pomegranates, watermelons, papaya

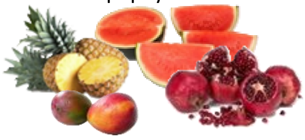

(1) (2) (3) (4) (5) (6)

BERRIES
e.g. strawberries, raspberries, blackberries

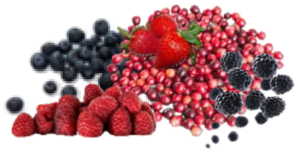

(1) (2) (3) (4) (5) (6)

BANANAS

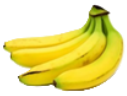

(1) (2) (3) (4) (5) (6)

APPLES AND PEARS

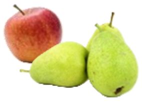

(1) (2) (3) (4) (5) (6)

AVOCADOS

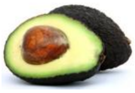

(1) (2) (3) (4) (5) (6)

OLIVES

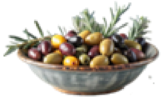

(1) (2) (3) (4) (5) (6)

DRIED FRUIT
e.g. apricots, plums, apples

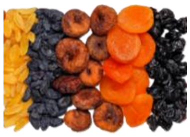

(1) (2) (3) (4) (5) (6)

SWEET FRUIT PRESERVES AND CANDIED FRUIT
e.g. jams, compotes

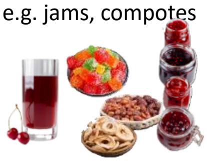

(1) (2) (3) (4) (5) (6)

Over the past 12 months, how often have you eaten:	Never or almost never	Once a month or less frequently	Several times a month	Several times a week	Every day	Several times a day
Consumption frequency codes	(1)	(2)	(3)	(4)	(5)	(6)
**Meat products and fish**						

WIENERS, LUNCHEON MEAT, HOT DOGS

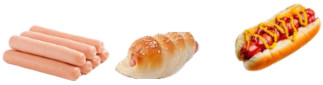

(1) (2) (3) (4) (5) (6)

SAUSAGES (VARIOUS TYPES)
e.g. sausage, kabanos sausage, bacon

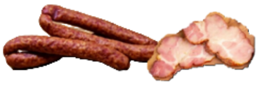

(1) (2) (3) (4) (5) (6)

HIGH-QUALITY COOKED MEATS
e.g. ham, pork fillet

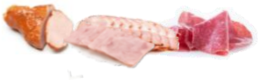

(1) (2) (3) (4) (5) (6)

CURED MEATS AND OFFAL PRODUCTS
e.g. black pudding, pâté, brawn

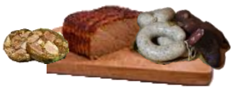

(1) (2) (3) (4) (5) (6)

RED MEATS
e.g. pork chop, frikadelle, steak, beef stew

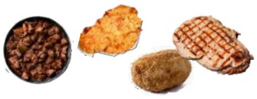

(1) (2) (3) (4) (5) (6)

POULTRY AND RABBIT MEAT
e.g. chicken, turkey, duck

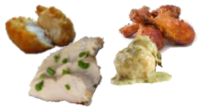

(1) (2) (3) (4) (5) (6)

GAME
e.g. wild boar, roe deer, hare, quail meat

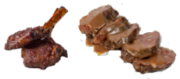

(1) (2) (3) (4) (5) (6)

LEAN FISH
e.g. cod, pollock, tuna, blue grenadier, zander

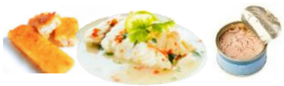

(1) (2) (3) (4) (5) (6)

OILY FISH
e.g. salmon, mackerel, herring, sardines, carp, eel

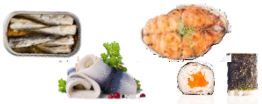

(1) (2) (3) (4) (5) (6)

Over the past 12 months, how often have you eaten:	Never or almost never	Once a month or less frequently	Several times a month	Several times a week	Every day	Several times a day
Consumption frequency codes	(1)	(2)	(3)	(4)	(5)	(6)
**Vegetables and seeds/nuts**						

CRUCIFEROUS VEGETABLES<br>e.g. cabbage, cauliflower, broccoli, kale<br>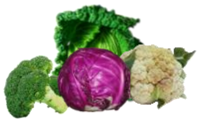<br>(1) (2) (3) (4) (5) (6)	YELLOW AND ORANGE-COLOURED VEGETABLES<br>e.g. carrot, sweet pepper<br>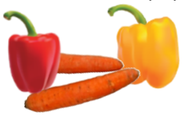<br>(1) (2) (3) (4) (5) (6)	GREEN LEAFY VEGETABLES<br>e.g. lettuce, rocket, spinach, celery<br>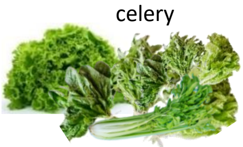<br>(1) (2) (3) (4) (5) (6)	TOMATOES<br>e.g. raspberry tomatoes, cherry tomatoes, lima tomatoes<br>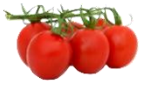<br>(1) (2) (3) (4) (5) (6)
VEGETABLES SUCH AS<br>e.g. cucumber, pumpkin, zucchini, aubergine<br>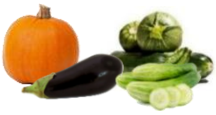<br>(1) (2) (3) (4) (5) (6)	ROOT AND OTHER VEGETABLES<br>e.g. parsley root, beetroots, mixed vegetable salads<br>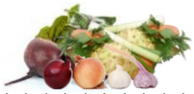<br>(1) (2) (3) (4) (5) (6)	FRESH LEGUME SEEDS<br>e.g. maize, French beans, peas<br>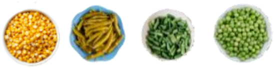<br>(1) (2) (3) (4) (5) (6)	DRIED LEGUME SEEDS<br>e.g. broad beans, beans, chickpeas<br>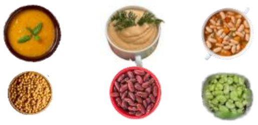<br>(1) (2) (3) (4) (5) (6)
POTATOES<br>e.g. boiled, baked<br>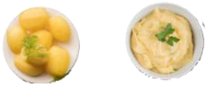<br>(1) (2) (3) (4) (5) (6)	FRENCH FRIES, POTATO PANCAKES<br>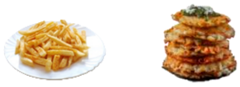<br>(1) (2) (3) (4) (5) (6)	POTATO DISHES<br>e.g. potato dumplings, Silesian dumplings<br>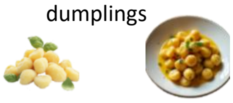<br>(1) (2) (3) (4) (5) (6)	NUTS<br>e.g. peanuts, walnuts, hazelnuts, almonds, pistachios<br>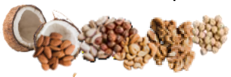<br>(1) (2) (3) (4) (5) (6)
NUT SPREADS<br>e.g. peanut butter, chocolate and nut spread<br>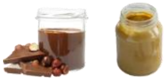<br>(1) (2) (3) (4) (5) (6)	SEEDS<br>e.g. pumpkin seeds, sunflower seeds, sesame seeds<br>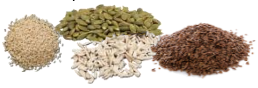<br>(1) (2) (3) (4) (5) (6)		

Over the past 12 months, how often have you drunk:	Never or almost never	Once a month or less frequently	Several times a month	Several times a week	Every day	Several times a day
Consumption frequency codes	(1)	(2)	(3)	(4)	(5)	(6)
**Drinks**						

FRUIT JUICES AND NECTARS<br>e.g. orange juice, apple nectar<br>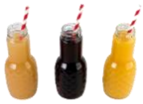<br>(1) (2) (3) (4) (5) (6)	VEGETABLE AND VEGETABLE-FRUIT JUICES<br>e.g. tomato juice, carrot juice, carrot and orange juice<br>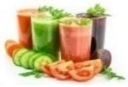<br>(1) (2) (3) (4) (5) (6)	ENERGY DRINKS<br>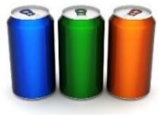<br>(1) (2) (3) (4) (5) (6)	SWEETENED DRINKS (SPARKLING AND STILL)<br>e.g. cola-type drinks, sweetened tea<br>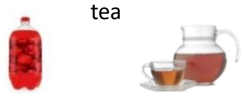<br>(1) (2) (3) (4) (5) (6)
WATER<br>e.g. tap water, spring water, mineral water, sparkling/still water<br>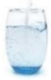<br>(1) (2) (3) (4) (5) (6)	LEMONADE and so-called FLAVOURED WATER<br>e.g. strawberry-flavoured, apple-flavoured<br>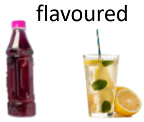<br>(1) (2) (3) (4) (5) (6)		

The images in the questionnaire were used in accordance with the Freepik and Pixabay licences valid at the time of download.

Table S2. Components of the pro-Healthy Diet Index for children aged 7-9 years old (pHDI)

No.	Components of the pHDI (consumption frequency for 1 point)	Items of the FFQ
1	Fruit	Stone fruit
	(≥ several times a day, i.e. ≥ 2.00 times/day)	Kiwi and citrus fruit
		Tropical fruits
		Berries
		Bananas
		Apples and pears
		Avocados
		Olives
2	Vegetables	Cruciferous vegetables
	(≥ several times a day, i.e. ≥ 2.00 times/day)	Yellow and orange-coloured vegetables
		Green leafy vegetables
		Tomatoes
		Vegetables such as e.g. cucumber, pumpkin
		Root and other vegetables
		Fresh legume seeds
3	Pulses (≥ several times a week, i.e. ≥ 0.571 times/day)	Dried legume seeds
4	Nuts and seeds	Nuts
	(≥ several times a week, i.e. ≥ 0.571 times/day)	Seeds
5	Wholegrain cereals	Wholemeal breads
	(≥ daily, i.e. ≥ 1.00 times/day)	Coarse grain groats, unrefined
		Breakfast cereals
		Wholegrain pasta (dishes)
6	Unsweetened dairy products	Milk and natural milk drinks
	(≥ several times a day, i.e. ≥ 2.00 times/day)	Natural curs cheeses
7	Fish	Lean fish
	(≥ several times a week, i.e. ≥ 0.571 times/day)	Oily fish
8	Vegetable oils (≥ several times a week, i.e. ≥ 0.571 times/day)	Oil

Table S3. Components of the non-Healthy Diet Index for children aged 7-9 years old (nHDI)

No.	Components of the nHDI (consumption frequency for 1 point)	Items of the FFQ
1	Sugar and sweets (> several times a week, i.e. > 0.571 times/day)	Sugar for sweetening drinks<br>Honey for sweetening dishes and drinks<br>Chocolate, chocolate candies and bars<br>Non-chocolate candies<br>Biscuits an cakes<br>Ice creams and custard
2	Salty snacks (> several times a week, i.e. > 0.571 times/day)	Salty snacks
3	Sweetened dairy products (> several times a week, i.e. > 0.571 times/day)	Sweetened milk and milk drinks<br>Flavoured smooth cheese
4	Ready-to-eat breakfast cereals (> several times a week, i.e. > 0.571 times/day)	Ready-to-eat breakfast cereals products
5	Sweet fruit preserves and candied fruits (> several times a week, i.e. > 0.571 times/day)	Dried fruit<br>Sweet fruit preserves and candied fruit
6	Fast-food (> several times a month, i.e. > 0.1 times/day)	Fast food dishes<br>French fries, potato pancakes
7	Processed meat (> several times a week, i.e. > 0.571 times/day)	Weners, luncheon, meat, hot dogs<br>Sausages (various types)<br>High-quality cooked meats<br>Cured meats and offal products
8	Sweetened beverages (> several times a month, i.e. > 0.1 times/day)	Sweetened drinks (sparkling and still)<br>Lemonade and so-call flavoured water
9	Energy drinks (never or almost never, i.e. 0 times/day)	Energy drinks

Table S4. Internal reliability of the pro-Healthy Diet Index for children aged 7-9 years old (pHDI) (n=77)

No.	Components of the pHDI	Cronbach's alpha*
	pHDI	**0.62**
1	Fruit	0.59
2	Vegetables	0.56
3	Pulses	0.60
4	Nuts and seeds	0.65
5	Wholegrain cereals	0.53
6	Unsweetened dairy products	0.58
7	Fish	0.57
8	Vegetable oils	0.65

*Cronbach's alpha values for the pHDI after excluding a given component

Table S5. Internal reliability of the non-Healthy Diet Index for children aged 7-9 years old (nHDI) (n=77)

No.	Components of the nHDI	Cronbach's alpha*
	nHDI	**0.51**
1	Sugar and sweets	0.49
2	Salty snacks	0.46
3	Sweetened dairy products	0.51
4	Ready-to-eat breakfast cereals	0.49
5	Sweet fruit preserves and candied fruits	0.49
6	Fast food	0.55
7	Processed meat	0.45
8	Sweetened beverages	0.42
9	Energy drinks	0.48

*Cronbach's alpha values for the nHDI after excluding a given component
